# Comparative Analysis of the COPD Assessment Test and Modified DECAF Score in Predicting Clinical Outcomes Among Patients With Acute Exacerbation of COPD: A Prospective Observational Study

**DOI:** 10.7759/cureus.79605

**Published:** 2025-02-25

**Authors:** Darshini V, Govindasaami Vinod, Hariprasad Balakrishnan

**Affiliations:** 1 Pulmonology, Sri Ramachandra Institute of Higher Education and Research, Chennai, IND

**Keywords:** chronic obstructive pulmonary disease, disease progression, hospital mortality, length of stay, risk assessment, severity of illness index, treatment outcome

## Abstract

Background

Acute exacerbation of chronic obstructive pulmonary disease (AECOPD) significantly impacts patient outcomes and healthcare resources. While various assessment tools exist, the comparative effectiveness of the COPD Assessment Test (CAT) and Dyspnea, Eosinopenia, Consolidation, Acidemia, and Atrial Fibrillation (DECAF) score in predicting clinical outcomes remains unclear. This study aims to determine whether the modified DECAF score provides superior prognostic accuracy compared to the CAT in predicting clinical outcomes of patients with AECOPD.

Methodology

A prospective observational study was conducted at the Department of Respiratory Medicine, Sri Ramachandra Institute of Higher Education and Research, from January 2023 to April 2024. The study enrolled 120 participants aged 40-80 years with physician-diagnosed COPD, using consecutive sampling. Patients with stable, well-controlled comorbidities and no exacerbations within 90 days were included. Data collection involved demographic information, clinical parameters, and laboratory investigations using standardized protocols. The CAT score assessed symptom burden across eight parameters, while the modified DECAF score evaluated dyspnea, eosinopenia, consolidation, acidemia, and atrial fibrillation. Statistical analysis employed Fisher's exact test and ANOVA, with p<0.05 considered significant.

Results

The study population comprised 104 (86.7%) male patients and 16 (13.3%) female patients, with 45 (37.5%) participants aged 61-70 years. The modified DECAF score demonstrated superior prognostic capability compared to CAT scores. In CAT score stratification, among patients with scores <10, 35 (79.54%) achieved cure while nine (20.45%) died; in the 10-20 category, 40 (86.95%) were cured and six (13.04%) died; and for scores >20, 23 (76.66%) recovered while seven (23.33%) died, showing no significant association (p = 0.474). In contrast, the modified DECAF system showed that all 70 (100%) low-risk and 21 (100%) intermediate-risk patients survived, while in the high-risk category, seven (24.13%) were cured and 22 (75.86%) died (p = 0.001). Ventilatory support requirements significantly increased with higher modified DECAF risk categories, with 26 (89.65%) high-risk patients requiring invasive mechanical ventilation (p = 0.001). The mean hospital stay duration varied significantly across modified DECAF categories: low-risk 7.27 days, intermediate-risk 8.33 days, and high-risk 17.43 days (p = 0.001).

Conclusion

The modified DECAF score demonstrates superior prognostic capability compared to CAT in predicting AECOPD outcomes, showing significant associations with mortality, ventilatory support requirements, and hospital stay duration. These findings support incorporating modified DECAF scoring into routine clinical practice for improved risk stratification and targeted interventions in AECOPD management.

## Introduction

Chronic obstructive pulmonary disease (COPD) represents a significant global health burden, characterized by persistent respiratory symptoms and airflow limitation due to airway and/or alveolar abnormalities. This condition affects approximately 400 million individuals worldwide, with projections indicating a substantial increase in prevalence through 2050 [1]. Acute exacerbations of COPD (AECOPD) constitute critical events in the natural history of the disease, often necessitating hospitalization and contributing significantly to morbidity, mortality, and healthcare expenditure [2,3].

The identification of patients at elevated risk during AECOPD episodes remains paramount for optimal clinical management and resource allocation. Various prognostic tools have emerged to address this crucial need, among which the COPD Assessment Test (CAT) and the Dyspnea, Eosinopenia, Consolidation, Acidemia, and Atrial Fibrillation (DECAF) score have gained particular prominence [4,5]. The CAT score, developed as a multidimensional assessment tool, evaluates the impact of COPD on patient health status through eight items covering various symptomatic domains. This instrument has demonstrated substantial utility in both stable COPD and during exacerbations, offering insights into disease severity and quality of life implications [6,7].

Conversely, the DECAF score emerged as a specific prognostic tool for AECOPD, incorporating five key clinical parameters to predict in-hospital mortality [5]. Subsequent validation studies have confirmed its robust predictive capability across diverse clinical settings [8,9]. The score has demonstrated particular utility in identifying low-risk patients suitable for home treatment, potentially reducing unnecessary hospitalizations and healthcare costs [10,11].

The pathophysiological basis for AECOPD involves complex interactions between environmental triggers and host responses, leading to increased airway inflammation and physiological deterioration [12,13]. These exacerbations significantly impact disease progression, with frequent episodes associated with accelerated decline in lung function and increased mortality risk [14,15]. The accurate prediction of outcomes during these episodes becomes crucial for implementing appropriate interventions and optimizing resource utilization [16].

Recent comparative analyses of prognostic tools in AECOPD have yielded varying results regarding their relative efficacy [17,18]. While both the CAT and DECAF scores offer valuable prognostic information, their comparative utility in predicting specific outcomes such as length of stay, need for ventilatory support, and short-term mortality remains incompletely characterized [19,20]. The CAT score's strength lies in its comprehensive assessment of symptomatic burden and functional impact [21], whereas the DECAF score's advantage stems from its incorporation of objective physiological parameters and a simplified scoring system [5,21].

Understanding the relative merits of these scoring systems in predicting various inpatient outcomes holds significant clinical implications. Such knowledge could facilitate more informed decision-making regarding admission, level of care, and resource allocation. Therefore, this study aims to determine whether the modified DECAF score provides superior prognostic accuracy compared to the CAT in predicting clinical outcomes of patients with AECOPD.

## Materials and methods

Study design and setting

A prospective observational study was conducted in the Department of Respiratory Medicine at Sri Ramachandra Institute of Higher Education and Research, a tertiary care teaching hospital in Porur, Chennai, India. The facility features a dedicated respiratory medicine unit with 60 beds, including a 10-bed respiratory intensive care unit equipped with advanced ventilatory support systems. The study utilized the department's comprehensive diagnostic facilities, including a spirometry laboratory, arterial blood gas analysis, and radiological services, ensuring standardized assessment of all study parameters.

Study period

The study was conducted from January 2023 to April 2024, encompassing a 16-month duration to ensure adequate sample recruitment and follow-up.

Ethics committee approval

The study protocol received approval from the Institutional Ethics Committee of Sri Ramachandra Institute of Higher Education and Research (approval number: CSP-MED/22/DEC/81/150). The research adhered to the principles of the Declaration of Helsinki and the Indian Council of Medical Research guidelines for biomedical research. Written informed consent was obtained from all participants in their preferred language, with provisions for legally authorized representatives in cases of severe respiratory distress. The consent process included a detailed explanation of study procedures, potential risks and benefits, and participants' right to withdraw without affecting their standard of care.

Sample size estimation

The sample size was calculated using Cochran's formula: $n = \frac{Z^2 \times p \times (1-p)}{d^2}$ where Z = 1.96 for 95% confidence level, p = prevalence, and d = absolute precision. Based on the study by Bansal et al. (2020), which reported a mortality rate of 60.71% with DECAF scores >3 in their cohort of 116 patients, we used p = 0.6071 [21]. With an absolute precision of 10% and a 95% confidence interval, the minimum required sample size was calculated as 120 participants.

Sampling method

Consecutive sampling was employed to minimize selection bias while ensuring temporal representation across the study period. This non-probability sampling technique was deemed appropriate given the study's objectives and setting, as it allowed systematic recruitment of all eligible patients presenting with AECOPD during the specified timeframe. The method's advantages included reduced sampling bias compared to convenience sampling, enhanced feasibility in the acute care setting, and better representation of seasonal variations in COPD exacerbations.

Inclusion and exclusion criteria

Patients aged 40-80 years with physician-diagnosed COPD, graded according to GOLD spirometry criteria, were included. The study required participants to have stable, well-controlled comorbidities and no exacerbations within the previous 90 days. Patients with terminal illnesses were excluded. The study also excluded non-COPD respiratory exacerbations, uncontrolled comorbidities, patients unwilling for admission, and those too sick to perform the CAT score assessment.

Data collection procedure

A dedicated research fellow, trained in respiratory medicine, conducted all data collection to ensure consistency and accuracy. The investigator underwent specialized training in administering the CAT questionnaire and assessing dyspnea using the e-MRC scale. The primary investigator personally administered all questionnaires and scoring tools, maintaining standardized assessment conditions across participants.

Demographic data, clinical parameters, and laboratory investigations were systematically recorded using a structured proforma. Clinical assessment included documentation of respiratory symptoms (dyspnea, cough with expectoration, wheeze), presence of fever, and radiological findings. Functional status was evaluated using the extended Medical Research Council (e-MRC) dyspnea scale. Laboratory parameters, including absolute eosinophil count, were measured using standardized hospital protocols. The data collection tools underwent pilot testing with 10 patients to ensure feasibility and clarity.

The investigator conducted daily follow-up of all enrolled patients, documenting clinical progress, ventilatory support modifications, and complications. Quality control measures included weekly data verification by a senior pulmonologist and regular monitoring of data completeness and accuracy.

Study tools

CAT

The CAT score, developed and validated by Jones et al. (2009), represents a comprehensive patient-reported outcome measure encompassing eight distinct domains: cough frequency and severity, sputum production, chest tightness, breathlessness during activities, activity limitations, confidence in leaving home, sleep quality, and energy levels. Each domain employs a semantic 6-point differential scale ranging from 0 to 5, culminating in a total score range of 0 to 40. The instrument demonstrates robust psychometric properties, with documented internal consistency (Cronbach's α = 0.88) and test-retest reliability (ICC = 0.8). Score interpretation follows clinically validated thresholds, where scores below 10 indicate low impact, 10-20 represent medium impact, and scores exceeding 20 signify high impact on health status [4].

Extended MRC Dyspnea Scale

The e-MRC dyspnea scale, based on the original scale validated by Bestall et al. (1999), grades breathlessness from 1-5, with additional subdivisions (5a, 5b) for severe dyspnea [22]. This tool assesses functional limitations due to breathlessness during daily activities.

DECAF and Modified DECAF Score

The DECAF scoring system, initially validated by Steer et al. (2012) in their seminal study of 920 patients, integrates five critical clinical parameters: dyspnea severity assessed through e-MRC criteria, eosinopenia defined as counts below 0.05×10⁹/L, radiological evidence of consolidation, acidemia characterized by pH below 7.3, and the presence of atrial fibrillation [5]. The modified DECAF score enhances the original framework by implementing a weighted scoring approach that stratifies patients into three distinct risk categories. This modification, validated through subsequent research by Echevarria et al. (2016), assigns particular emphasis to severe acidemia (pH<7.25) and incorporates extended dyspnea grading. The resultant risk stratification delineates low risk (0-1 points), intermediate risk (2 points), and high risk (≥3 points), providing clinicians with a more refined prognostic tool for acute care decision-making [9].

Data analysis

The gathered data was analyzed using IBM SPSS Statistics for Windows, Version 26.0 (released 2019; IBM Corp., Armonk, New York, United States). Descriptive statistics were presented as frequencies, percentages, means, and standard deviations. The association between CAT scores and clinical outcomes was analyzed using Fisher's exact test. Analysis of variance (ANOVA) was employed to assess the relationship between hospital stay duration and both CAT and modified DECAF scores. The predictive accuracy of both scoring systems for mortality and ventilatory support requirements was evaluated using Fisher's exact test. A p-value <0.05 was considered statistically significant. The analysis specifically focused on comparing the predictive capability of CAT and modified DECAF scores for duration of hospitalization, ventilatory support requirements, and mortality outcomes.

## Results

Table 1 shows the age and gender distribution of study participants. The study included a total of 120 participants, with a predominant male representation of 104 (86.7%) compared to 16 (13.3%) female participants. The age distribution revealed that the majority of participants were in the 61-70 years age group, comprising 45 (37.5%) individuals, followed by 30 (25.0%) participants in the 71-80 years age group. Twenty-one (17.5%) participants were above 80 years of age, while 17 (14.2%) were between 51 and 60 years. The smallest age group was under 50 years, with only seven (5.8%) participants.

**Table 1:** Age and gender distribution of study participants

Age and gender distribution		Frequency (N=120)	Percent
Age group	< 50 years	7	5.8
	51 - 60 years	17	14.2
	61 - 70 years	45	37.5
	71 - 80 years	30	25.0
	> 80 years	21	17.5
Gender	Male	104	86.7
	Female	16	13.3

Table 2 shows the clinical characteristics and laboratory parameters of study participants. Among the 120 study participants, respiratory symptoms were predominant, with shortness of breath being the most frequently reported symptom affecting 114 (95.0%) individuals. Cough with expectoration was present in 99 (82.5%) participants, while wheeze was reported by 36 (30.0%) participants. Fever was observed in 21 (17.5%) cases, and radiological evidence of consolidation was present in 5 (4.2%) participants. Regarding functional status, the extended Medical Research Council (e-MRC) dyspnea scale showed an equal distribution between grade 5 and 5a, each comprising 44 (36.7%) participants, while 32 (26.7%) were classified as grade 5b. A significant proportion of participants, 45 (37.5%), experienced frequent hospitalizations (≥2 per year). The mean duration of hospital stays was 8.22 ± 5.54 days. Laboratory investigation revealed a mean absolute eosinophil count of 214.59 ± 293.86 cells/µL, suggesting varying degrees of eosinophilic inflammation among the study population.

**Table 2:** Clinical characteristics and laboratory parameters of study participants e-MRC: Extended Medical Research Council

Clinical characteristics and laboratory parameters		Frequency (N=120)	Percent
Fever	Yes	21	17.5
	No	99	82.5
Cough with expectoration	Yes	99	82.5
	No	21	17.5
Wheeze	Yes	36	30.0
	No	84	70.0
Shortness of breath	Yes	114	95.0
	No	6	5.0
Consolidation	Yes	5	4.2
	No	115	95.8
e-MRC dyspnea grading	5	44	36.7
	5a	44	36.7
	5b	32	26.7
Frequency of hospitalization (≥2 / year)	Yes	45	37.5
	No	75	62.5
Hospital stay duration in days (mean ± S.D)		8.22 ± 5.54	
Absolute Eosinophil Count (cells/µL)		214.59 ± 293.86	

**Figure 1:**
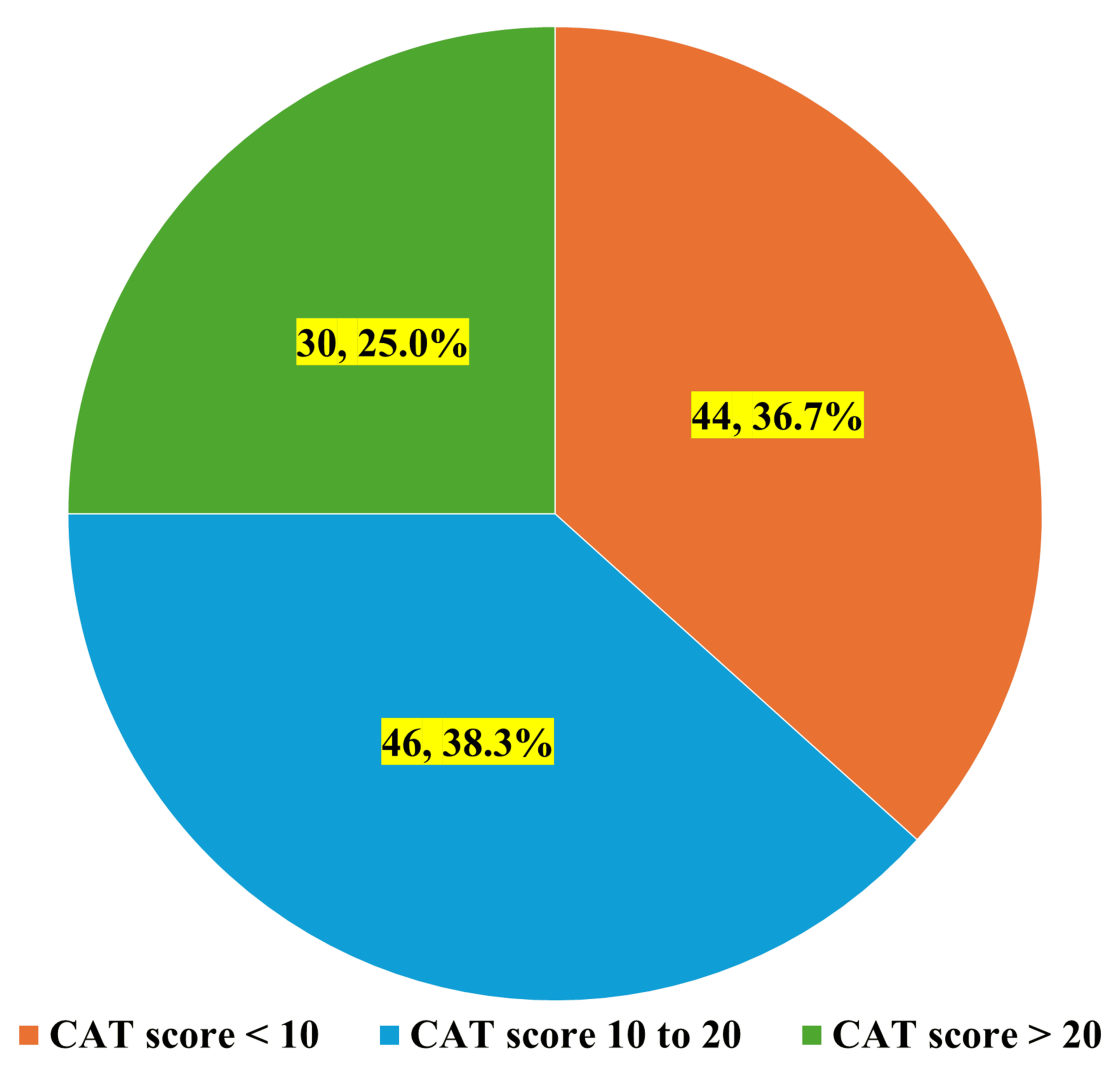
Distribution of CAT (COPD Assessment Test) scores among study participants COPD: Chronic obstructive pulmonary disease

Figure 1 shows the distribution of CAT scores among study participants. The largest proportion comprised participants with moderate CAT scores (10 to 20), accounting for 46 (38.3%) individuals. This was followed by those with low CAT scores (<10), representing 44 (36.7%) participants. The smallest group consisted of patients with high CAT scores (>20), comprising 30 (25.0%) individuals. This distribution suggests that the majority of the study population (74 participants, 61.7%) experienced moderate to high impact of COPD on their daily lives, as indicated by CAT scores ≥10, which is considered clinically significant in COPD management and prognostication.

**Figure 2:**
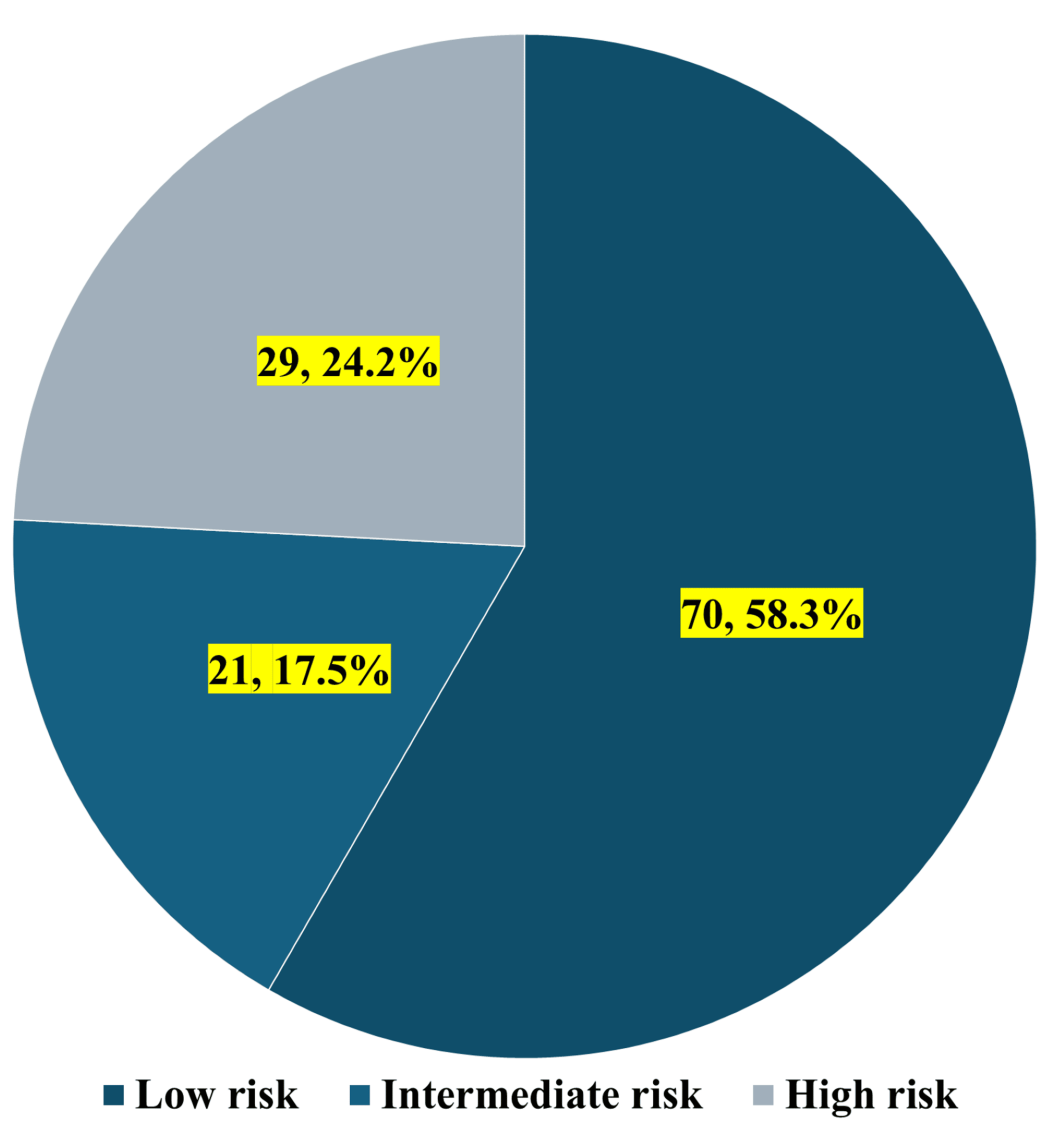
Risk stratification of study participants based on the modified DECAF score DECAF: Dyspnea, Eosinopenia, Consolidation, Acidemia, and Atrial Fibrillation

Figure 2 shows the risk stratification of study participants based on the modified DECAF score. The majority of participants were classified as low-risk, comprising 70 (58.3%) individuals. The intermediate-risk category included 21 (17.5%) participants, while the high-risk group consisted of 29 (24.2%) subjects. This distribution pattern suggests that while the majority of the study population demonstrated favorable prognostic indicators, a considerable proportion (approximately 41.7%) exhibited moderate to high-risk characteristics based on the modified DECAF scoring criteria, warranting careful clinical monitoring and potentially more aggressive therapeutic interventions.

**Figure 3:**
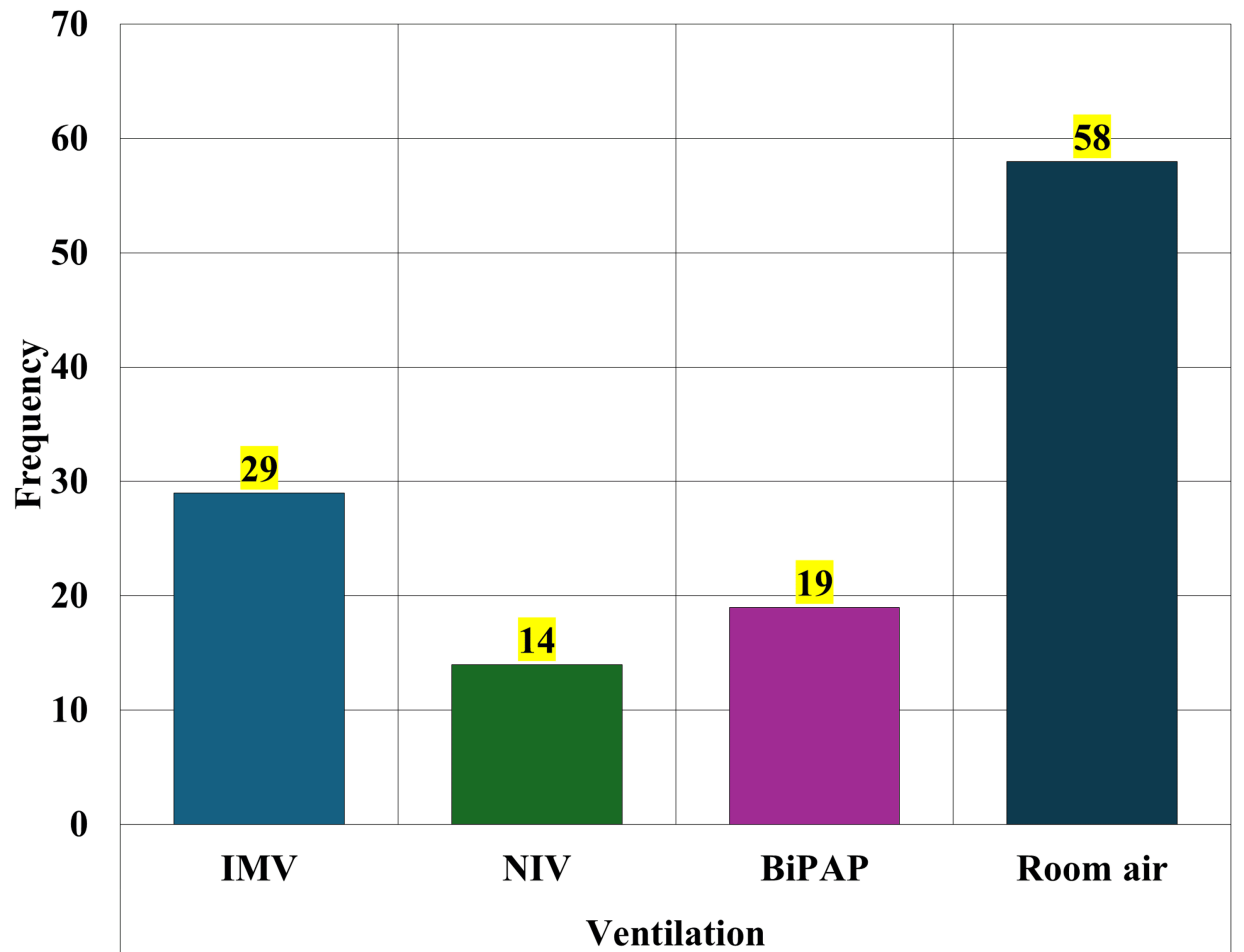
Distribution of ventilatory support modalities among study participants IMV: Invasive mechanical ventilation; BiPAP: Bi-level positive airway pressure; NIV: Non-invasive ventilation

Figure 3 shows the distribution of ventilatory support modalities among study participants. Room air was sufficient for the majority of participants, accounting for 58 (48.3%) cases. Invasive mechanical ventilation (IMV) was required in 29 (24.2%) patients, representing the second most common intervention. Bi-level positive airway pressure (BiPAP) support was necessary for 19 (15.8%) individuals, while non-invasive ventilation (NIV) was utilized in 14 (11.7%) cases.

**Figure 4:**
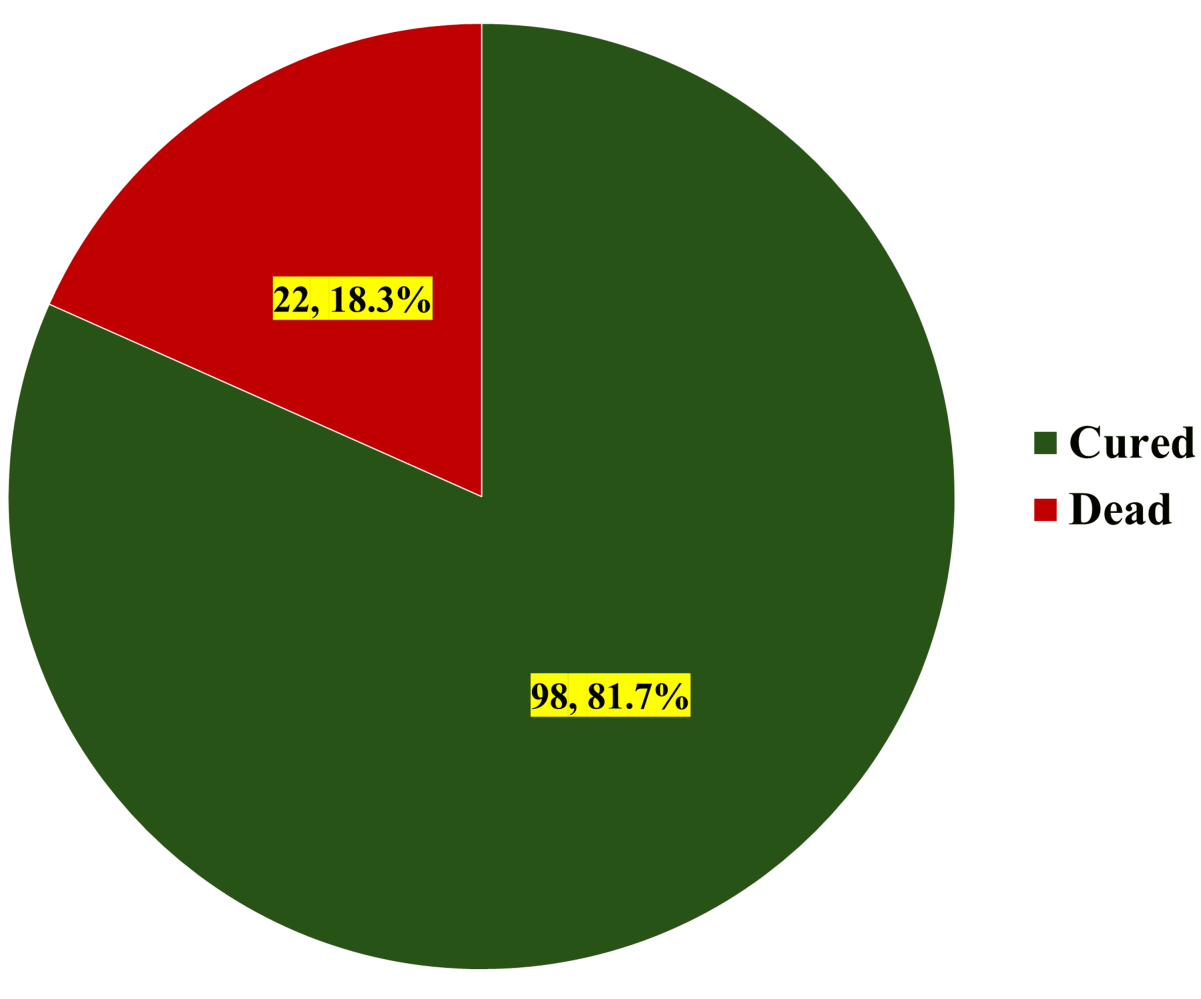
Clinical outcomes of study participants

Figure 4 shows the clinical outcomes of study participants. Analysis of clinical outcomes demonstrated that among the total study cohort (N = 120), a significant majority achieved clinical recovery, with 98 (81.7%) participants being successfully cured. However, there was notable mortality observed in the study population, with 22 (18.3%) patients experiencing fatal outcomes.

Table 3 shows the association of hospital stay duration with CAT scores and modified DECAF risk categories. Among the 98 survivors, the CAT score categorization showed minimal variation in the hospital stay duration across all three groups. Notably, patients with higher CAT scores did not demonstrate prolonged hospitalization periods. In contrast, the modified DECAF risk stratification revealed a clear progressive pattern in hospital stay duration. High-risk patients required significantly longer hospitalizations, with mean stays more than twice that of low-risk patients. This marked difference in the hospital stay duration across DECAF risk categories demonstrates its superior utility in predicting hospitalization length.

**Table 3:** Association of hospital stays duration with CAT scores and modified DECAF risk categories One-way analysis of variance (ANOVA) was used to compare mean hospital stay duration across CAT score categories and modified DECAF risk groups. Post hoc Tukey's HSD test was performed for significant ANOVA results. Levene's test was used to verify the homogeneity of variances. ^*^Statistically significant at p-value less than 0.05. CAT: COPD Assessment Test; DECAF: Dyspnea, Eosinopenia, Consolidation, Acidemia, and Atrial Fibrillation; COPD: Chronic obstructive pulmonary disease

Hospital stay duration vs CAT scores and modified DECAF risk categories		Hospital stay duration (days)			F value	ANOVA p-value
		N (98)	Mean	S.D.		
CAT score	< 10	35 (35.7%)	8.29	5.76	0.080	0.923
	10 to 20	40 (40.8%)	8.4	5.26		
	> 20	23 (23.5%)	7.83	5.87		
Modified DECAF score	Low risk	70 (71.4%)	7.27	4.03	13.842	0.001^*^
	Intermediate risk	21 (21.4%)	8.33	6.31		
	High risk	7 (7.2)	17.43	8.14		

Table 4 shows the association between ventilatory support requirements and clinical severity indicators. The analysis revealed distinct patterns in ventilatory support needs across different severity indicators. For CAT score stratification, the requirement for invasive mechanical ventilation was most pronounced in the high CAT score group (12 patients, 40%). However, the most striking observation emerged from the modified DECAF risk categorization. In the high-risk group, 26 patients (89.7%) required invasive mechanical ventilation, while the majority of low-risk patients (54 patients, 77.1%) maintained adequate oxygenation on room air. The intermediate-risk group showed a predominant need for non-invasive ventilation methods, with 17 patients (80.9%) requiring either NIV or BiPAP support.

**Table 4:** A cross-tabulation analysis of association between ventilatory support requirements and clinical severity indicators (CAT score and modified DECAF score) Fisher's exact test was used instead of the chi-square test due to expected cell frequencies <5 in multiple cells. ^*^Statistically significant at p-value less than 0.05 CAT: COPD Assessment Test; DECAF: Dyspnea, Eosinopenia, Consolidation, Acidemia, and Atrial Fibrillation; IMV: Invasive mechanical ventilation; BiPAP - Bi-level positive airway pressure; NIV: Non-invasive ventilation; COPD: Chronic obstructive pulmonary disease

Ventilatory Support Requirements versus Clinical Severity Indicators		Ventilation				Fischer’s exact value	p-value
		IMV (n = 29)	NIV (n = 14)	BiPAP (n = 19)	Room Air (n = 58)		
CAT score	< 10 (n = 44)	10 (22.7%)	3 (6.8%)	5 (11.4%)	26 (59.1%)	10.268	0.113
	10 to 20 (n = 46)	7 (15.2%)	7 (15.2%)	10 (21.8%)	22 (47.8%)		
	> 20 (n = 30)	12 (40%)	4 (13.3%)	4 (13.3%)	10 (33.4%)		
Modified DECAF score	Low risk (n = 70)	3 (4.3%)	5 (7.1%)	8 (11.4%)	54 (77.1%)	125.237	0.001^*^
	Intermediate risk (n = 21)	0 (0%)	8 (38.1%)	9 (42.9%)	4 (19.0%)		
	High risk (n = 29)	26 (89.7%)	1 (3.4%)	2 (6.9%)	0 (0%)		

Table 5 shows the association between CAT and modified DECAF scores with clinical outcomes. The study revealed contrasting patterns in outcome prediction between the two scoring systems. The CAT score demonstrated relatively consistent cure rates across all categories, with successful outcomes ranging from 76.66% to 86.95%. In contrast, the modified DECAF score showed remarkable discriminative ability in predicting outcomes. Both low and intermediate-risk categories achieved 100% cure rates (91 patients), while the high-risk category experienced significantly poorer outcomes, with only seven patients (24.13%) achieving cure. This stark contrast in outcome prediction highlights the modified DECAF score's superior prognostic capability.

**Table 5:** Association between CAT and modified DECAF Scores with clinical outcomes in acute exacerbation of COPD patients (N=120) ^*^Fisher's exact test was used and statistically significant at p-value less than 0.05 CAT: COPD Assessment Test; DECAF; Dyspnea, Eosinopenia, Consolidation, Acidemia, and Atrial Fibrillation

CAT and modified DECAF scores versus clinical outcomes		Outcome		Fischer’s exact value	p-value
		Cured (n = 98)	Dead (n = 22)		
CAT score	< 10 (n = 44)	35 (79.54%)	9 (20.45%)	1.492	0.474
	10 to 20 (n = 46)	40 (86.95%)	6 (13.04%)		
	> 20 (n = 30)	23 (76.66%)	7 (23.33%)		
Modified DECAF score	Low risk (n = 70)	70 (100%)	0 (0%)	84.532	0.001^*^
	Intermediate risk (n = 21)	21 (100%)	0 (0%)		
	High risk (n = 29)	7 (24.13%)	22 (75.86%)		

Table 6 shows the association between DECAF and modified DECAF risk stratification in acute exacerbations of COPD patients. The comparative analysis between traditional and modified DECAF scoring systems revealed important reclassification patterns. The majority of traditionally classified low-risk patients (70 patients) maintained their risk category in the modified system. However, a significant shift was observed in the intermediate-risk group, where 25 patients (69.5%) were reclassified as high-risk under the modified system. This reclassification pattern suggests that the modified DECAF score may provide a more conservative risk assessment, potentially leading to earlier identification of high-risk patients requiring intensive intervention.

**Table 6:** Association between DECAF and modified DECAF risk stratification in acute exacerbation of COPD patients (N=120) *Fisher's exact test was used and statistically significant at p-value less than 0.05 CAT: COPD Assessment Test; DECAF; Dyspnea, Eosinopenia, Consolidation, Acidemia, and Atrial Fibrillation

DECAF score	Modified DECAF score			Fischer’s exact value	p-value
	Low risk (n = 70)	Intermediate risk (n = 21)	High risk (n = 29)		
Low risk (n = 82)	70 (85.4%)	10 (12.2%)	2 (2.4%)	88.930	0.001^*^
Intermediate risk (n = 36)	0 (0%)	11 (30.5%)	25 (69.5%)		
High risk (n = 2)	0 (0%)	0 (0%)	2 (100%)		

## Discussion

This comprehensive investigation yields substantive insights regarding the comparative prognostic utility of CAT and modified DECAF scores in acute exacerbations of COPD. Our findings demonstrate the modified DECAF score's superior predictive capability across multiple clinical endpoints, particularly regarding mortality, ventilatory support requirements, and hospitalization duration.

The demographic composition of our study cohort, predominantly male (86.7%) with concentration in the 61-70 year age group (37.5%), corresponds with established epidemiological patterns. Echevarria et al. (2016) reported comparable demographic distributions in their validation cohort of 880 patients [9], while Zidan et al. (2020) observed similar patterns in their comparative analysis of 100 patients [23]. This consistency enhances the external validity of our findings within the broader COPD population context.

The prevalence of respiratory symptoms in our cohort, particularly dyspnea (95.0%) and productive cough (82.5%), aligns with the symptomatological patterns described by Ritchie and Wedzicha (2020) in their comprehensive review of COPD exacerbations [12]. This symptomatic pattern underscores the significant impact of exacerbations on respiratory function and patient well-being, as elucidated by Wedzicha et al. (2014) in their analysis of acute COPD exacerbations [3]. The significant proportion of patients in this study experiencing frequent hospitalizations (37.5% with ≥2 per year) corroborates findings from Donaldson et al. (2002) regarding the impact of recurrent exacerbations on disease progression [15].

Our analysis revealed the modified DECAF score's superior discriminative capability in predicting clinical outcomes compared to the CAT score. This finding substantiates the work of Steer et al. (2012), who initially validated the DECAF score with an area under the receiver operating characteristic (ROC) curve of 0.86 for in-hospital mortality prediction [5]. The robust stratification observed in our study, particularly the 75.86% mortality rate in high-risk patients versus zero mortality in low and intermediate-risk groups, parallels the findings of Nafae et al. (2015) and Bansal and Gaude (2020) in their respective cohorts [21,24].

The significant association between modified DECAF scores and ventilatory support requirements (p = 0.001) represents a crucial finding. The observation that 89.65% of high-risk patients required invasive mechanical ventilation while 77.1% of low-risk patients maintained adequate oxygenation on room air aligns with findings from Echevarria et al. (2018) regarding risk-stratified intervention requirements [10]. This pattern has substantial implications for resource allocation and clinical decision-making.

The CAT score, while demonstrating utility in symptom burden assessment, showed limited correlation with acute clinical outcomes in our study. This finding diverges somewhat from Tu et al. (2014) and Feliz-Rodriguez et al. (2013), who reported significant associations between CAT scores and exacerbation severity [7,19]. However, their focus on outpatient outcomes and longer-term follow-up may explain this discrepancy. This finding contrasts with observations by Kelly et al. (2012), who reported significant associations between CAT scores and disease severity in stable COPD patients [25].

The mortality patterns observed in our study (18.3% overall) demonstrated a remarkable correlation with modified DECAF risk categories, with 75.86% mortality in the high-risk group compared to zero mortality in low and intermediate-risk groups. This robust stratification capability corroborates findings from Memon et al. (2019), who reported similar mortality distributions across DECAF risk categories in their cohort of 125 patients [26].

The length of hospital stay analysis revealed significant associations with modified DECAF risk categories (p = 0.001), with high-risk patients demonstrating substantially longer hospitalizations (mean 17.43 ± 8.14 days) compared to low-risk patients (7.27 ± 4.03 days). This pattern aligns with findings from Yadavilli et al. (2016), who documented similar correlations between DECAF scores and hospitalization duration [27].

The superior prognostic performance of the modified DECAF score may be attributed to its incorporation of objective physiological parameters, as suggested by Echevarria et al. (2019) in their comparative analysis of early warning scores [18]. The integration of acidemia and consolidation parameters provides direct indicators of physiological derangement, potentially explaining its enhanced predictive capability compared to the symptom-focused CAT score.

Our findings regarding the reclassification patterns between traditional and modified DECAF scores demonstrate substantial concordance while revealing important risk redistribution, particularly in the intermediate-risk category. This observation supports the refinements introduced in the modified scoring system, as documented by Zidan et al. (2020) in their comparative analysis of prognostic tools [23].

The clear delineation of risk categories in our study has important implications for clinical decision-making, supporting the findings of Unal et al. (2023) regarding the utility of risk stratification tools in emergency department settings [28]. The ability to accurately identify high-risk patients early in their presentation enables more targeted resource allocation and intervention strategies, potentially improving outcomes through early optimization of care pathways.

These findings contribute significantly to the evolving body of evidence regarding prognostic tools in AECOPD management, as outlined in recent systematic reviews by Ji et al. (2023) [29]. The superior performance of the modified DECAF score across multiple outcome measures suggests its potential role as a preferred risk stratification tool in acute care settings, particularly when decisions regarding the level of care and resource allocation are paramount.

Clinical significance

The findings of this study have substantial implications for clinical practice in the management of AECOPD. The superior prognostic accuracy of the modified DECAF score in predicting mortality, ventilatory support requirements, and hospital stay duration provides clinicians with a reliable tool for early risk stratification and resource allocation.

The clear delineation of risk categories (low, intermediate, and high) facilitates evidence-based decision-making regarding admission, level of care, and intensity of monitoring. As demonstrated by Nadeem et al. (2021), this stratification can potentially streamline patient care pathways and optimize healthcare resource utilization [11]. The absence of mortality in low and intermediate-risk groups suggests that these patients might be candidates for less intensive care settings or early discharge protocols, aligning with findings from Echevarria et al. (2018) regarding safe home treatment for selected patients [10].

The strong association between modified DECAF scores and ventilatory support requirements provides valuable guidance for early intervention planning. This predictive capability enables proactive preparation for potential mechanical ventilation needs, particularly in high-risk patients, potentially improving outcomes through earlier intervention. Furthermore, the correlation with length of stay helps in better hospital resource planning and communication with patients and families regarding expected hospitalization duration.

Strengths of the study

This study's primary strengths lie in its comprehensive assessment of multiple clinical outcomes and the direct comparison of two widely used scoring systems. The inclusion of a substantial sample size (120 patients) with strict inclusion/exclusion criteria enhanced the reliability of our findings. The study's prospective design minimized recall bias and allowed for accurate data collection across all parameters.

The incorporation of multiple outcome measures, including mortality, ventilatory support requirements, and length of stay, provides a holistic evaluation of prognostic tool effectiveness. Additionally, the use of standardized assessment tools and objective clinical parameters strengthens the validity of our findings.

Limitations

The single-center nature of the study may limit generalizability to other healthcare settings or geographical regions. The predominance of male participants (86.7%) might not fully represent the disease patterns in female COPD patients. The exclusion of patients too sick to perform the CAT score potentially introduced selection bias, possibly underestimating the prevalence of severe cases. Additionally, the study's focus on in-hospital outcomes precluded assessment of long-term follow-up data, which might provide valuable insights into the predictive value of these scoring systems for post-discharge outcomes.

Recommendations

Future research should include multi-center studies with more diverse patient populations to validate these findings across different healthcare settings. Long-term follow-up studies are needed to assess the predictive value of these scoring systems for post-discharge outcomes. Implementation studies evaluating the integration of modified DECAF scoring into clinical decision support systems could enhance its practical utility. Development of automated scoring systems could facilitate wider adoption in clinical practice.

## Conclusions

This study demonstrates the superior prognostic capability of the modified DECAF score compared to the CAT score in predicting inpatient outcomes among AECOPD patients. The modified DECAF score showed significant associations with mortality, ventilatory support requirements, and hospital stay duration, providing a reliable tool for clinical decision-making. While the CAT score remains valuable for assessing symptom burden, its utility in predicting acute outcomes appears limited. The clear stratification of risk categories using the modified DECAF score enables more effective resource allocation and treatment planning. These findings support the incorporation of modified DECAF scoring into routine clinical practice for AECOPD patients, potentially improving patient care through better risk stratification and more targeted interventions.
